# Cornell Alliance for Science Evaluation of Consensus on Genetically Modified Food Safety: Weaknesses in Study Design

**DOI:** 10.3389/fpubh.2017.00079

**Published:** 2017-04-12

**Authors:** Michael N. Antoniou, Claire J. Robinson

**Affiliations:** ^1^Gene Expression and Therapy Group, Faculty of Life Sciences & Medicine, Department of Medical and Molecular Genetics, King’s College London, Guy’s Hospital, London, UK; ^2^GMWatch, Norwich, UK

**Keywords:** Cornell Alliance for Science, citizen science, genetically modified foods, glyphosate, scientific consensus, genetically modified food health risks

## Abstract

Cornell Alliance for Science has launched an initiative in which “citizen scientists” are called upon to evaluate studies on health risks of genetically modified (GM) crops and foods. The purpose is to establish whether the consensus on GM food safety claimed by the American Association for the Advancement of Science (AAAS) is supported by a review of the scientific literature. The Alliance’s citizen scientists are examining more than 12,000 publication abstracts to quantify how far the scientific literature supports the AAAS’s statement. We identify a number of fundamental weaknesses in the Alliance’s study design, including evaluation is based only on information provided in the publication abstract; there is a lack of clarity as to what material is included in the 12,000 study abstracts to be reviewed, since the number of appropriately designed investigations addressing GM food safety are few; there is uncertainty as to whether studies of toxic effects arising from GM crop-associated pesticides will be included; there is a lack of clarity regarding whether divergent yet equally valid interpretations of the same study will be taken into account; and there is no definition of the cutoff point for consensus or non-consensus on GM food safety. In addition, vital industry proprietary biosafety data on GM crops and associated pesticides are not publicly available and is thus cannot inform this project. Based on these weaknesses in the study design, we believe it is questionable as to whether any objective or meaningful conclusion can be drawn from the Alliance’s initiative.

## Consensus on Genetically Modified (GM) Food Safety?

Cornell Alliance for Science has launched an initiative in which “citizen scientists” are called upon to evaluate studies on health risks of GM crops and foods (1).

The background to the initiative is that in 2012 the board of the American Association for the Advancement of Science (AAAS) issued the following statement:

Consuming foods containing ingredients derived from GM crops is no riskier than consuming the same foods containing ingredients from crop plants modified by conventional plant improvement techniques.

However, as the Alliance explains, others have denied that any consensus on the safety of GM foods exists. In 2013, the European Network of Scientists for Social and Environmental Responsibility (ENSSER) issued a statement, which criticized the 2012 AAAS statement and asserted: “We strongly reject claims… that there is a ‘scientific consensus’ on GMO safety and that the debate on this topic is ‘over’” (2).

The purpose of the Alliance’s initiative is to establish whether the claimed consensus on GM food safety is supported by a review of the scientific literature. The project aims to perform this task using a similar methodology to that employed by Cook and colleagues in their 2013 study of the climate change literature. This study concluded that 97% of the peer-reviewed literature supported the consensus on the existence of human-caused climate change (3).

In order to address this question for the similarly contested proposed consensus on GM food safety, the Alliance is examining more than 12,000 publication abstracts (1996–2015) available from the Web of Science. The aim is to quantify, using these abstracts, how far the scientific literature supports or does not support the AAAS statement on the consensus on GM food safety.

In principle, this is a laudable initiative that aims to address an important public health question. However, in our view, some aspects of the methodology give rise to concerns that deserve to be addressed. If they are not addressed, we believe that the initiative risks failing to meet its stated objectives.

## The Alliance’s Methodology

We have been informed by Jaron Porciello, Associate Director for Research Data and Engagement at the Alliance for Science, that the abstracts included in the citizen scientists’ review were selected in the following way. A total of 12,000 abstracts were chosen from approximately 144,000 using the Web of Science database. After 6 months of testing, keywords were selected that were informed by exploring other meta-analyses (critical, supportive, and neutral regarding GM foods). Tests were run to analyze what was lost when using one word over another, and, where overlap exists, by consulting references such as the UN Food and Agriculture Organization’s AGROVOC and MEDLINE in order to ensure that the broadest possible concepts were covered.

The website of the Alliance for Science further explains, “Each abstract will be rated twice, by two independent raters (and no rater will receive the same ‘set’ of abstracts to rate), and once again by the author of the abstract (pending their participation).”

## Concerns about the Methodology

We believe that there are a number of problems with the Alliance’s approach.

First, each reviewed publication will only be judged as to its significance purely from the abstract. However, the message of a study lies in the fine detail of its results and their various interpretations. This is especially the case with many studies on GM food health risks. Frequently, the authors conclude that there were no treatment-related adverse effects in the GM-fed groups of animals, but a close reading of the detail of the study reveals indications of toxicity or signs of toxicity in the GM-fed animals.

For example, a Monsanto-sponsored 90-day rat feeding study with the company’s GM Bt insecticidal maize MON863 concluded that it was as safe and nutritious as the non-GM control maize (4). However, a reanalysis of the full published results in combination with the complete raw dataset, undertaken by a team of academic scientists working independently of the industry, revealed adverse effects or signs of potential toxicity, especially pertaining to liver and kidney function, in the GM-fed animals (5).

Monsanto responded by dismissing these statistically significant and potentially adverse effects as “unrelated to treatment or of no biological or clinical importance because they failed to demonstrate a dose-response relationship, reproducibility over time, association with other relevant changes (e.g., histopathology), occurrence in both sexes, difference outside the normal range of variation, or biological plausibility with respect to cause-and-effect” (6).

This type of dismissal is contrary to normal scientific practice, which calls for statistically significant biological differences caused by an intervention to be followed up with further research in order to determine their long-term consequences with respect to health.

As another example, a three-generation feeding study in rats with GM Bt insecticidal maize reported in the abstract that there were “some minimal histopathological changes in liver and kidney” in the GM-fed animals (7). These changes were described as “minor” in a much-cited review of GM food safety studies by Snell and colleagues (8). Yet examination of the detail of the study reveals that the GM-fed rats suffered damage to their liver and kidneys and alterations in blood biochemistry, which some scientists may view as unresolved safety questions demanding further study.

These examples suggest that statistically significant changes in GM-fed animals can either be viewed as unimportant or as indications that further research is needed to understand their mechanism and significance, depending on the individual viewpoints of the authors and/or reviewers.

These examples also illustrate that it is necessary to have full access to (minimally) the full results section of a publication and that conclusions about the safety of a GM food cannot be derived purely from the information provided in the abstract.

## Few Long-Term Studies on Health Implications of GM Foods

The number of properly designed and executed long-term studies looking at health implications of GM foods are very few. A commercial lifespan feeding study in pigs under real farm conditions found that animals fed a mixture of commercialized GM crops (soy and maize) resulted in elevated levels of severe stomach inflammation and heavier uteri in females, compared with controls fed a non-GM diet (9).

In another example, in 2012, a study was published that found liver and kidney damage in rats fed glyphosate-tolerant GM maize NK603 and low doses of its associated herbicide Roundup over a 2-year period (10).

The study gave rise to a great deal of controversy. In response, the French food safety agency ANSES conducted a search for other comparable long-term laboratory animal feeding studies on GM herbicide-tolerant crops. It found only two (11). One was a two-year study in mice by Malatesta and colleagues, which found more pronounced signs of liver aging in the GM soy-fed group (12). The other was a study that found “no apparent adverse effect in rats” fed GM soybeans (13). However, in this latter study, the fact that glyphosate was only detected at the level of quantification (0.1 ppm) in the GM soy implies that, contrary to usual farming practice, this crop was not sprayed with this herbicide during cultivation, since it is well established that relatively high residues of glyphosate are routinely found in US-grown soy (14, 15).

Given the results of ANSES’s search, it is unclear how 12,000 study abstracts with direct relevance to health have been identified. This raises the question of which types of publication will be included in the review. Will only publications describing original research be evaluated, or will reviews of the literature also be included?

This is an essential consideration because it is important not to take at face value the conclusions of reviews of studies, but instead to examine the results of the original studies covered by the reviews. This is because the conclusions of reviews can be marred by bias and omissions.

For instance, Snell and colleagues published a review of animal feeding studies with GM foods (8). Some of these studies showed toxic effects in the GM-fed animals. This included Malatesta and colleagues’ study showing more pronounced liver aging in the animals fed GM soy (12). However, Snell and colleagues dismissed these effects as being of “no biological or toxicological significance” on the grounds of various methodological weaknesses (8)—in spite of the fact that studies concluding safety for the GM food tested suffered from the same inadequacies in study design (16).

## GM Crop-Associated Herbicide Residues as a Source of Toxicity

Multiple sources of potential harm from GM food consumption are acknowledged and covered in the scientific literature. Toxic effects in principle could arise directly from the GM transformation process, resulting from disturbed gene function leading to altered biochemistry. Alternatively, toxicity could arise from increased exposures to the pesticides that are used in GM crop cultivation. Around 85% of GM crops are engineered to withstand application of herbicides (17), which in the majority of cases are glyphosate-based products such as Roundup.

Thus the question arises as to whether studies that examine the toxic effects of glyphosate will also be included in the Alliance’s review. This question is not addressed by the Alliance’s website and was not answered by Jaron Porciello in his email communications with the authors. However, it is important as animals and humans will inevitably be exposed to high levels of residues in food made from glyphosate-tolerant GM crops (14, 15, 18, 19) and these may pose health risks in their own right (20–22).

By the same token, will studies looking at toxicity from Bt toxin in a non-GM-related context be included? Although these studies are not on GM crops, they are relevant to a discussion of GM crop toxicity since large numbers of these crops are engineered to systemically express this protein (17).

## Varying Interpretations of Datasets

Experimental datasets are subject to more than one interpretation, that is, different and perhaps even divergent yet equally valid interpretations of results can be arrived at by different scientists. This is inherent in the nature of the scientific exercise and an essential driver of scientific discourse. To illustrate this point in the context of the Alliance’s initiative, statistically significant differences in physiological parameters arising from the consumption of a GM food compared to its non-GM control can be viewed by some scientists as biologically not relevant/significant and thus an indication of safety, while other scientists may see such differences as signs of possible toxicity that need to be followed up with additional research. Thus conclusions of safety arrived at by the authors can frequently be open to challenge.

The only way that the Alliance’s citizen scientist reviewers can confirm the validity of the authors’ conclusions is to have access to the whole study dataset. Restricting the evaluation of a study simply to the scrutiny of a given publication’s abstract does not meet this crucial requirement and thus introduces a high level of risk that the citizen scientists’ exercise will fail to meet its stated objectives.

It is, therefore, open to question as to whether the Alliance can derive any meaningful conclusions by having the citizen scientists look only at the abstracts. It is unclear if the citizen scientists and the reading public will be made aware of these major limitations of the exercise.

## Transparency

We also have concerns about the transparency of the methodology. According to Jaron Porciello in email communications with the authors, the full dataset, including all the selected and tested keywords and search strings, will only be made available upon conclusion of the study. However, this is unacceptable as it denies observers the opportunity to constructively critique the methodology with the aim of ensuring scientific rigor. From an objective standpoint, it is a concern that the methodology has not been made fully available at the outset. This may raise suspicion among the skeptical public who form the target audience for this exercise that the criteria upon which the abstracts are evaluated might be retrospectively selected to fit a preordained conclusion.

## Missing Industry Proprietary Data

The study of GM food safety is undermined by the fact that the GM seed developer and pesticide companies own the biosafety studies that they conduct on their products to support regulatory approval. Frequently, the data from these investigations are kept hidden as commercial secrets and not published in the peer review literature. In addition, scientists working outside of the industry lack access to the necessary research materials, that is, the GM crop under examination and its non-GM isogenic closest relative, grown under the same conditions.

A review addressing these issues stated that confidential business information (CBI) is often claimed for documentation and materials supporting the biosafety assessments of GMOs intended for environmental release and food, and feed use, but “such claims oftentimes marginally serve their legitimate purpose to protect commercial interests and unnecessarily limit transparency and public peer review of data submitted to regulatory authorities.” The author added that CBI and proprietary claims also restrict access to transgene sequence data, GM seeds, and other GMO materials, which “precludes the development of independent research and monitoring strategies.”

The author concluded that such claims “hinder the accumulation of biosafety data in the open, peer-reviewed literature, which is needed for both public and scientific consensus-building on safety issues and for improvements to the risk-assessment procedure itself” (23).

These vital biosafety data thus are not available to inform projects such as the Alliance’s initiative, which are designed to make judgments on GM food safety.

## Cutoff Point for Consensus

It is unclear at which cutoff point the organizers of the Alliance’s initiative will conclude on a “consensus” on GM food safety. From the point of view of protecting public health, even if 90% of the studies reviewed conclude in favor of safety and 10% do not, this should be sufficient to prove a lack of consensus. By analogy, if a new aircraft type is tested and only 10% of the tests show a problem, it is clear that those 10% of test results should not be dismissed in favor of the 90% of results demonstrating safety.

## Conclusion

In this commentary, we have highlighted weaknesses in the study design of the Cornell Alliance for Science’s citizen scientist initiative to evaluate the scientific literature pertaining to GM food safety. Amongst these shortcomings are:

1. Evaluation is based only on information provided in the abstract of any given publication, even though the full impact of the GM diet is only revealed by a close reading of the study’s complete dataset.
2. It is unclear what material is included in the 12,000 study abstracts to be reviewed by the citizen scientists, since the number of appropriately designed investigations directly addressing GM food safety are very few.
3. It is unclear whether studies of actual and potential toxic effects arising from GM crop-associated pesticides (for example, glyphosate and Bt toxin) will be included in the review.
4. Different scientists can interpret the same results in different yet equally valid ways, with some concluding safety while others see potential or actual harm. This again highlights the need to examine the full dataset of any given publication to arrive at a conclusion of either safety or harm. If such differing interpretations of the same dataset still exist, then this necessitates a conclusion of non-consensus on GM food safety.
5. The cutoff point for consensus or non-consensus on GM food safety has not been defined from the outset.

Based on the above weaknesses in the study design, it is questionable as to whether any objective or meaningful conclusion can be drawn from the Alliance’s exercise.

## Author Contributions

This article was conceived by MA. Both authors contributed equally to the drafting of the manuscript.

## Conflict of Interest Statement

MA declares no conflict of interest. CR is editor at GMWatch, a public information and news service that urges caution in the use of genetic engineering in food and agriculture.
